# Supplementary material for: Heparin-based hydrogel scaffolding alters the transcriptomic profile and increases the chemoresistance of MDA-MB-231 triple-negative breast cancer cells
Source: Biomater Sci. 2020 Feb 13;8(10):2786–96. doi: 10.1039/c9bm01481k (PMC7497406; doi:10.1039/c9bm01481k)
Supplement: Supplementary file 2 [file BM-008-C9BM01481K-s002.zip › Supplementary File 4/EGFvControl/Pathways/my_analysis.Gsea.1545200981068/HALLMARK_PEROXISOME.html]

Details for gene set HALLMARK\_PEROXISOME[GSEA]

|  || Dataset | expr.class.cls#EGF\_versus\_CONTROL.class.cls#EGF\_versus\_CONTROL\_repos |
| Phenotype | class.cls#EGF\_versus\_CONTROL\_repos |
| Upregulated in class | CONTROL |
| GeneSet | HALLMARK\_PEROXISOME |
| Enrichment Score (ES) | -0.20323116 |
| Normalized Enrichment Score (NES) | -0.87552416 |
| Nominal p-value | 0.7037037 |
| FDR q-value | 0.77575433 |
| FWER p-Value | 1.0 |
Table: GSEA Results Summary

  

Fig 1: Enrichment plot: HALLMARK\_PEROXISOME      
 Profile of the Running ES Score & Positions of GeneSet Members on the Rank Ordered List

  

| PROBE | DESCRIPTION (from dataset) | GENE SYMBOL | GENE\_TITLE | RANK IN GENE LIST | RANK METRIC SCORE | RUNNING ES | CORE ENRICHMENT || 1 | SLC25A19 | na |  |  | 33 | 2.800 | 0.0430 | No |
| 2 | CLN6 | na |  |  | 1082 | 1.485 | 0.0119 | No |
| 3 | NUDT19 | na |  |  | 1341 | 1.395 | 0.0206 | No |
| 4 | PRDX1 | na |  |  | 1452 | 1.363 | 0.0367 | No |
| 5 | ACSL4 | na |  |  | 1621 | 1.312 | 0.0488 | No |
| 6 | STS | na |  |  | 1789 | 1.271 | 0.0604 | No |
| 7 | GNPAT | na |  |  | 1828 | 1.261 | 0.0785 | No |
| 8 | ELOVL5 | na |  |  | 1901 | 1.246 | 0.0947 | No |
| 9 | ITGB1BP1 | na |  |  | 2109 | 1.194 | 0.1029 | No |
| 10 | DHCR24 | na |  |  | 2137 | 1.190 | 0.1205 | No |
| 11 | TOP2A | na |  |  | 2151 | 1.189 | 0.1388 | No |
| 12 | ECI2 | na |  |  | 2215 | 1.174 | 0.1543 | No |
| 13 | ABCD3 | na |  |  | 2448 | 1.124 | 0.1601 | No |
| 14 | ERCC3 | na |  |  | 2881 | 1.047 | 0.1542 | No |
| 15 | SLC23A2 | na |  |  | 2916 | 1.041 | 0.1690 | No |
| 16 | CNBP | na |  |  | 2934 | 1.036 | 0.1847 | No |
| 17 | MSH2 | na |  |  | 3641 | 0.915 | 0.1623 | No |
| 18 | YWHAH | na |  |  | 3864 | 0.875 | 0.1647 | No |
| 19 | ISOC1 | na |  |  | 4105 | 0.836 | 0.1655 | No |
| 20 | SLC25A4 | na |  |  | 4552 | 0.770 | 0.1545 | No |
| 21 | FDPS | na |  |  | 4955 | 0.713 | 0.1448 | No |
| 22 | ABCC5 | na |  |  | 5023 | 0.704 | 0.1526 | No |
| 23 | ACAA1 | na |  |  | 5086 | 0.695 | 0.1604 | No |
| 24 | ACSL1 | na |  |  | 5124 | 0.689 | 0.1695 | No |
| 25 | HRAS | na |  |  | 5618 | 0.623 | 0.1536 | No |
| 26 | ALDH9A1 | na |  |  | 5781 | 0.599 | 0.1547 | No |
| 27 | EHHADH | na |  |  | 5911 | 0.581 | 0.1573 | No |
| 28 | CLN8 | na |  |  | 6611 | 0.490 | 0.1285 | No |
| 29 | GSTK1 | na |  |  | 7100 | 0.433 | 0.1099 | No |
| 30 | SIAH1 | na |  |  | 7187 | 0.424 | 0.1122 | No |
| 31 | PEX5 | na |  |  | 7498 | 0.385 | 0.1021 | No |
| 32 | MLYCD | na |  |  | 7518 | 0.383 | 0.1072 | No |
| 33 | SOD1 | na |  |  | 7945 | 0.332 | 0.0902 | No |
| 34 | IDE | na |  |  | 8196 | 0.305 | 0.0820 | No |
| 35 | LONP2 | na |  |  | 8419 | 0.282 | 0.0749 | No |
| 36 | IDH1 | na |  |  | 8504 | 0.271 | 0.0749 | No |
| 37 | NR1I2 | na |  |  | 8778 | 0.239 | 0.0644 | No |
| 38 | SLC25A17 | na |  |  | 8824 | 0.235 | 0.0658 | No |
| 39 | PEX13 | na |  |  | 8966 | 0.216 | 0.0619 | No |
| 40 | ESR2 | na |  |  | 9094 | 0.202 | 0.0585 | No |
| 41 | ACOX1 | na |  |  | 9429 | 0.166 | 0.0436 | No |
| 42 | ERCC1 | na |  |  | 9755 | 0.129 | 0.0287 | No |
| 43 | IDI1 | na |  |  | 9872 | 0.119 | 0.0245 | No |
| 44 | HSD17B11 | na |  |  | 10030 | 0.103 | 0.0180 | No |
| 45 | SMARCC1 | na |  |  | 10226 | 0.078 | 0.0090 | No |
| 46 | VPS4B | na |  |  | 10468 | 0.057 | -0.0027 | No |
| 47 | SEMA3C | na |  |  | 10501 | 0.053 | -0.0035 | No |
| 48 | ABCB4 | na |  |  | 10527 | 0.051 | -0.0040 | No |
| 49 | SCP2 | na |  |  | 10592 | 0.039 | -0.0067 | No |
| 50 | FADS1 | na |  |  | 10812 | 0.012 | -0.0180 | No |
| 51 | RDH11 | na |  |  | 10948 | 0.003 | -0.0250 | No |
| 52 | PEX6 | na |  |  | 11097 | -0.017 | -0.0325 | No |
| 53 | CTBP1 | na |  |  | 11207 | -0.027 | -0.0378 | No |
| 54 | RETSAT | na |  |  | 12351 | -0.162 | -0.0950 | No |
| 55 | HSD11B2 | na |  |  | 12481 | -0.181 | -0.0988 | No |
| 56 | CRABP2 | na |  |  | 12999 | -0.243 | -0.1220 | No |
| 57 | PEX11B | na |  |  | 13277 | -0.286 | -0.1319 | No |
| 58 | TSPO | na |  |  | 13324 | -0.292 | -0.1297 | No |
| 59 | CDK7 | na |  |  | 13562 | -0.325 | -0.1369 | No |
| 60 | PEX2 | na |  |  | 13566 | -0.326 | -0.1318 | No |
| 61 | PEX11A | na |  |  | 14006 | -0.379 | -0.1488 | No |
| 62 | ACSL5 | na |  |  | 14462 | -0.439 | -0.1656 | No |
| 63 | HSD17B4 | na |  |  | 14809 | -0.499 | -0.1757 | No |
| 64 | FIS1 | na |  |  | 15335 | -0.571 | -0.1940 | Yes |
| 65 | CADM1 | na |  |  | 15381 | -0.579 | -0.1872 | Yes |
| 66 | HSD3B7 | na |  |  | 15423 | -0.585 | -0.1800 | Yes |
| 67 | SLC35B2 | na |  |  | 15492 | -0.595 | -0.1740 | Yes |
| 68 | CAT | na |  |  | 15662 | -0.617 | -0.1730 | Yes |
| 69 | ABCB9 | na |  |  | 15736 | -0.633 | -0.1667 | Yes |
| 70 | ECH1 | na |  |  | 16356 | -0.770 | -0.1868 | Yes |
| 71 | HMGCL | na |  |  | 16532 | -0.819 | -0.1829 | Yes |
| 72 | BCL10 | na |  |  | 16617 | -0.838 | -0.1739 | Yes |
| 73 | ABCD1 | na |  |  | 17179 | -1.003 | -0.1872 | Yes |
| 74 | CEL | na |  |  | 17224 | -1.014 | -0.1733 | Yes |
| 75 | ATXN1 | na |  |  | 17279 | -1.034 | -0.1596 | Yes |
| 76 | FABP6 | na |  |  | 17472 | -1.112 | -0.1519 | Yes |
| 77 | DLG4 | na |  |  | 17663 | -1.172 | -0.1431 | Yes |
| 78 | PEX14 | na |  |  | 17786 | -1.218 | -0.1301 | Yes |
| 79 | IDH2 | na |  |  | 17795 | -1.224 | -0.1110 | Yes |
| 80 | MVP | na |  |  | 17873 | -1.261 | -0.0948 | Yes |
| 81 | ACOT8 | na |  |  | 17936 | -1.295 | -0.0774 | Yes |
| 82 | CRAT | na |  |  | 18281 | -1.480 | -0.0718 | Yes |
| 83 | PRDX5 | na |  |  | 18957 | -2.467 | -0.0677 | Yes |
| 84 | DHRS3 | na |  |  | 18963 | -2.502 | -0.0280 | Yes |
| 85 | SOD2 | na |  |  | 18970 | -2.521 | 0.0119 | Yes |
Table: GSEA details [plain text format]

  

Fig 2: HALLMARK\_PEROXISOME      
 Blue-Pink O' Gram in the Space of the Analyzed GeneSet

  

Fig 3: HALLMARK\_PEROXISOME: Random ES distribution      
 Gene set null distribution of ES for **HALLMARK\_PEROXISOME**
